# Supplementary material for: Thermo- and pH-Responsive Gelatin/Polyphenolic Tannin/Graphene Oxide Hydrogels for Efficient Methylene Blue Delivery
Source: Molecules. 2021 Jul 27;26(15):4529. doi: 10.3390/molecules26154529 (PMC8347097; doi:10.3390/molecules26154529)
Supplement: Supplementary file 1 [file molecules-26-04529-s001.zip › molecules-1309806-supplementary.pdf]

Supplementary Material

Thermo- and pH-responsive gelatin/polyphenolic tannin/graphene oxide hydrogels for efficient methylene blue delivery

Ariel C. de Oliveira[1], Paulo R. Souza[1], Bruno H. Vilsinski[1,8], Manuel E. G. Winkler[1], Marcos L. Bruschi[7], Eduardo Radovanovic[4], Edvani C. Muniz[1,5], Wilker Caetano[6], Artur J. M. Valente[3]* and Alessandro F. Martins[1,2]*

[1] Group of polymeric materials and composites, department of chemistry, State University of Maringá (UEM), Maringá, PR 87020-900, Brazil: pg53548@uem.br (P.R.S); pg54114@uem.br (A.C.O); pg53546@uem.br (M.E.G.W)

[2] Laboratory of materials, macromolecules and composites, Federal University of Technology – Paraná (UTFPR), Apucarana, PR 86812-460, Brazil:afmartins@utfpr.edu.br (A.F.M)

[3] University of Coimbra, CQC, Department of Chemistry, 3004-535, Coimbra, Portugal: avalente@qui.uc.pt (A.J.M.V)

[4] Department of Chemistry, State University of Maringa, Maringá, Brazil: eradovanovic@uem.br (E.R)

[5] Department of Chemistry, Federal University of Piaui, Teresina, 64049-550 PI, Brazil: ecmuniz@uem.br (E.C.M)

[6] Núcleo de Pesquisas em Sistemas Fotodinâmicos, Chemistry Department, State University of Maringá, 87020-900 Maringá, PR, Brazil: wcaetano@uem.br (W.C)

[7] Laboratory of Research and Development of Drug Delivery Systems, Department of Pharmacy, State Univesity of Maringá (UEM), 5970 Colombo Avenue, 87020-900 Maringá, PR, Brazil: mlbruschi@uem.br (M.L.B)

[8] Chemistry Department, Institute of Exact Sciences, Federal University of Juiz de Fora, Juiz de Fora, MG 36036-900, Brazil: bhvilsinski2@uem.br (B.H.V).

Figure S1 shows that GE/TN/GO-MB hydrogels present no significant porosity difference ($p > 0.05$). However, the hydrogels loaded with MB present high porosity - between 64.54 and 77.26%. The porosity facilitates water diffusion toward the polymer matrix, favoring the solute release [1]. Indeed, porous and three-dimensional matrices can be used as drug delivery systems (DDSs) [2].

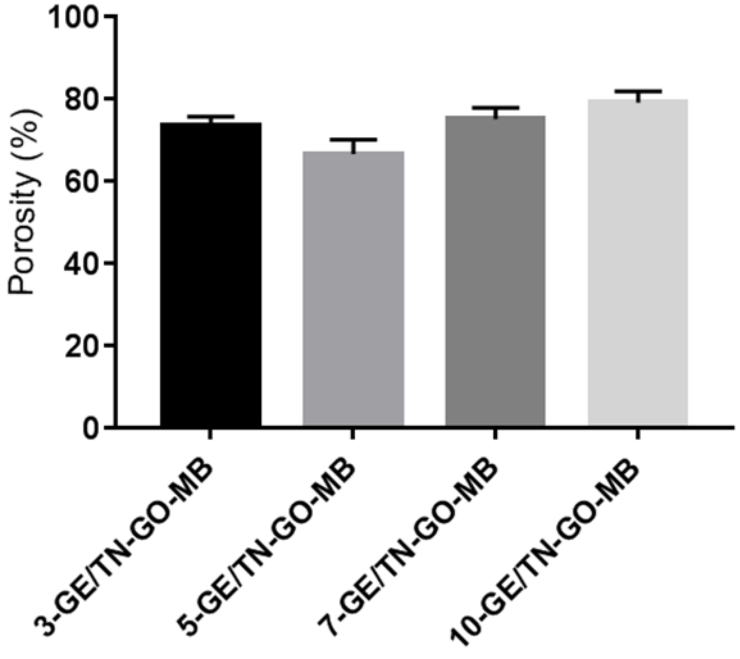

Figure S1. Porosity (%) of the [GE/TN/GO-MB hydrogels with different GO content (Table 1) measured at 37 ºC.

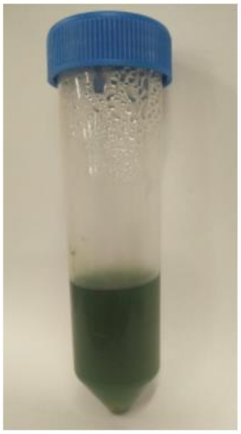

Figure S2. Digital images of a 10-GE/TN/GO-MB hydrogel after disintegration/dissolution (48 h) em SGF.

References

1. Berg, M.C., Zhai, L., Cohen, R.E., Rubner, M.F., 2006. Controlled Drug Release from Porous Polyelectrolyte Multilayers. Biomacromolecules 7, 357–364. https://doi.org/10.1021/bm050174e
2. Spizzirri, U.G., Cirillo, G. (Eds.), 2017. Functional Hydrogels in Drug Delivery: Key Features and Future Perspectives, 1st ed. CRC Press, Boca Raton, FL : CRC Press/ Taylor & Francis Group, 2017. https://doi.org/10.4324/9781315152271
